# Megakaryocytic Emperipolesis Associated with Thrombocytopenia: Causative or Coincidence?

**DOI:** 10.4274/tjh.2017.0211

**Published:** 2017-12-01

**Authors:** Manu Goyal, Sreeja Thandilath Thekkelakayil, Anurag Gupta

**Affiliations:** 1 AmPath Hyderabad Hospital, Clinics of Hematopathology and Molecular Hematopathology, Telangana, India; 2 AmPath Hyderabad Hospital, Clinic of Hematopathology, Telangana, India; 3 AmPath Hyderabad Hospital, Clinic of Cytogenetics, Telangana, India

**Keywords:** Thrombocytopenia, Megakaryocytic emperipolesis, GATA1

## To The Editor,

Phagocytosis, emperipolesis, and entosis are physiological and pathological phenomena characterized by the engulfment of one cell into another cell [1]. Emperipolesis is defined as active penetration of one cell by another, which remains intact [2]. Emperipolesis differs from phagocytosis in that an engulfed cell exists temporarily within another cell and with an intact normal structure, while in phagocytosis, the engulfed cell is destroyed by the proteolytic action of lysosomal enzymes [1,2]. Entosis is a non-apoptotic cell death mechanism that occurs in cell populations deprived of matrix attachment [3,4].

**Figure 1:**
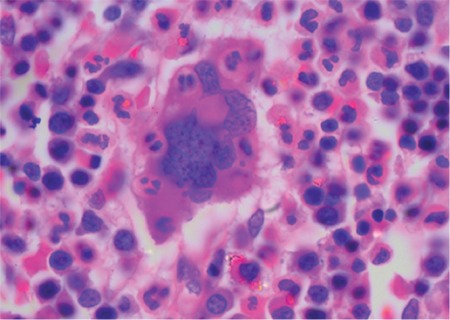
Photomicrograph of the trephine biopsy shows megakaryocytic emperipolesis containing neutrophils (hematoxylin and eosin stain, original magnification 630^x^).

A 31-year-old male presented with severe headache to the emergency department. He was afebrile without any organomegaly or neurological deficit. An urgent computed tomography scan of the brain showed subarachnoid hemorrhage. Complete blood counts revealed hemoglobin of 80 g/L, leukocyte count of 4.9x10^9^/L, platelet count of 5x10^9^/L, and a few giant platelets on peripheral smear. Prothrombin time, activated partial thromboplastin time, and fibrinogen were within the normal ranges. Bone marrow evaluation performed to assess the cause of severe thrombocytopenia showed normal erythropoiesis and myelopoiesis with increased megakaryocytes. These megakaryocytes showed neutrophils with marked emperipolesis (Figure 1). There was no evidence of malignancy or infiltrate. A working diagnosis of immune-mediated thrombocytopenia was issued and the patient was treated with steroids and intravenous immunoglobulins. In view of the marked thrombocytopenia and hemorrhagic complications, the patient was transfused with multiple units of single-donor platelets. Despite aggressive medical management, his platelet counts did not improve. He was discharged against medical advice and lost to follow-up.

Emperipolesis is a hallmark of Rosai-Dorfman disease (RDD); however, it can also be seen in both malignant hematolymphoid disorders (like Hodgkin lymphoma, non-Hodgkin lymphoma, acute myeloid leukemias, myeloproliferative disorders or myelodysplastic syndrome) and non-hematological malignancies (neuroblastoma, rhabdomyosarcoma) [1,5]. Emperipolesis can be either megakaryocytic or histiocytic. The former engulfs erythroblasts, myeloid cells, or neutrophils and is seen in hematolymphoid disorders, while the latter engulfs inflammatory cells (lymphocytes and plasma cells) as seen in RDD [1].

The exact mechanism for megakaryocytic emperipolesis is unknown. Centurione et al. [6] in their mice model suggested that abnormality in GATA1 transcription factor (either due to mutation or deletion) results in thrombocytopenia, megakaryocytic emperipolesis, and resultant myelofibrosis. Increased expression of P-selectin is known to mediate neutrophil sequestration on the outer surface of megakaryocytes, promoting increased neutrophil-megakaryocyte interactions [6,7]. A few studies indicated that the release of alpha-granular proteins, growth factors, and cytokines produced by megakaryocytes as well as neutrophil protease in the microenvironment induce emperipolesis [5,8]. The fate could be the cannibalism of the invading cell, host cell death, transcytosis, or division of both the invading and recipient cells [4,7]. Further research at the molecular level is needed to elucidate the underlying specific mechanisms.

With regards to platelet counts, there have been few case reports of megakaryocytic emperipolesis associated with thrombocytosis, rarely in thrombocytopenia associated with myelodysplasia and none associated with immune-mediated thrombocytopenia [9]. In the present case, whether megakaryocytic emperipolesis was responsible for the thrombocytopenia or simply a coincidence is difficult to establish. We present this rare phenomenon so that similar observations cumulatively would help in resolving this complex issue.
